# The Reality of Hypnotic Phenomena

**Published:** 1881-11

**Authors:** 


### THE REALITY OF HYPNOTIC PHENOMENA.

The *Lancet* publishes an article of Dr. Charles Richet considering the reality of the phenomena of hypnotism. It is impossible to fix upon a decisive test in this matter. We know that a fact is scientifically certain when the phenomenon, which is the evidence of it, can be reproduced at will by all persons who will use the same processes, as in the case of any chemical or physical manipulation. The phenomena of hypnotism are uncertain, intangible and variable; different persons, even though employing identical processes, are liable to obtain very different results. The only absolute sign possible is one's own experience, and that is applicable only to himself. There are, however, certain arguments which bear upon the case with almost, if not quite, the force of a demonstration.

1. It is absurd to suppose that all hypnotized persons have simulated sleep. Friends in whom we have absolute confidence may be among them; it is not possible to believe that they have conspired all at once to deceive us.

2. A close agreement has prevailed among certain of the phenomena of the manifestations for sixty years. "That would be a very strange simulation to be reproduced so frequently in so long a time with the same appearances—closed eyelids, fibrillar movements in the muscles of the face, hallucinations of vision and hearing, catalepsy, contracture"—and this among persons strangers to each other and who may be wholly ignorant of hypnotism.

3. Many of the phenomena can not be simulated without a profound knowledge of anatomy and physiology, which hardly any hypnotics possess. When the nerves of the hypnotized person are pressed the muscles supplied by them contract. Who among them knows what muscles should act under the influence of a particular nerve? Yet no mistake is made. "With somnambulists one can by direct incitation cause contraction of the muscles (rudimentary in man) moving the auricle of the ear. Now this contraction is impossible in the individual when awake." With a certain hysteric, who came under Dr. Richet's observation, "by opening the right eye aphasia was produced; while by opening the left eye no such effect was obtained. Certainly if this be simulation one must assume that the patient knows that speech is affected by the left cerebral hemisphere, and that the retina of the right eye is in relation with this hemisphere, while the right hemisphere is of no use for speech."

The hysterical contractures afford equally convincing evidence. "There is no individual strong enough to preserve voluntarily the contraction of a muscle during a quarter of an hour without one perceiving in it the slightest tendency to weakness or relaxation. Now somnambulists maintain their contractures for many hours, and on waking they have no recollection of, no fatigue from, this prolonged and improbable effort." Again, insensibility may be feigned ; "but how many persons are there who would have the courage to bear, without serious reason, pricks in the face, on the nostrils, or hands ; to allow their hair to be plucked out, and the conjunctiva, the nose, and the ears to be tickled ; to have pins thrust into the arms ; to drink nauseous liquids ; to breathe with delight ammonia or sulphurous acid?" Somnambulists oppose no resistance to tests like these. "Must we suppose that they exhibit heroism (and a very misplaced heroism) or anesthesia ?"—*Pop. Sc. Monthly.*

